# Making impressions count: An evaluation of the quality of information provided by orthodontic practices in London in response to the COVID-19 pandemic

**DOI:** 10.1016/j.heliyon.2020.e05516

**Published:** 2020-11-16

**Authors:** Julian Woolley, Christopher Donnell, Stuart Worthington

**Affiliations:** a Dental Core Trainee, King's College London, King's College Hospital NHS Foundation Trust, United Kingdom; b Dental Core Trainee, Newcastle Dental Hospital, Newcastle Upon Tyne NHS Hospitals Foundation Trust, United Kingdom; c Specialty Regsitrar, Dental Public Health, Public Health England, Newcastle upon Tyne, United Kingdom

**Keywords:** Social science, Orthodontic practice, COVID-19, Pandemic, Digital marketing, Ethical advertising, SARS-Cov-2

## Abstract

**Introduction/Objectives:**

As a result of the coronavirus disease 2019 (COVID-19) pandemic, primary care specialist orthodontic practices have been limited to providing emergency treatment only. This has resulted in a cessation of normal face-to-face services and patient advice can only be offered by remote means. A service evaluation was carried out to assess the quality of information published on websites and social media pages of specialist orthodontic practices in London, against General Dental Council guidance on communication and advertising and the British Orthodontic Society (BOS) COVID-19 specific guidance for orthodontics in primary care in relation to Coronavirus Disease 2019 (COVID-19) pandemic. This study also aimed to provide a gold standard template for orthodontic practices to aid in the delivery of information on a digital platform during the current (COVID-19) pandemic and future possible spikes.

**Materials and methods:**

All orthodontic practices providing care in the London region were identified from a CQC Database and subsequently checked against predetermined criteria based on the BOS guidance and the GDC Guidance on Ethical Advertising.

**Results:**

Of the 83 orthodontic practices sampled; 78 had a website of which 18 (23.1%) were non-compliant with GDC guidance. Facebook pages were identified for 62 orthodontic practices. 17 practices did not provide any update in relation to the COVID-19 pandemic. This was more frequently carried out on practice websites (78.2%) compared to Facebook pages (33.9%). A number of practices were identified as having novel strategies to manage communication during the COVID-19 pandemic.

**Conclusion:**

Variation was observed in information published by practices despite the regularly updated, blanket information provided by the BOS. Communication may have been delivered by a different means during the pandemic which this study did not account for. In addition, the sampling method may not have identified all practices within the London region, however the sample size seems appropriate to draw meaningful conclusions. The checklist created should help improve the delivery of future information.

## 1 Introduction

As of 29^th^ August 2020, more than 24.5 million cases and 832,879 deaths worldwide due to coronavirus disease 2019 (COVID-19) have been reported [1]. This novel form of severe acute respiratory syndrome coronavirus 2 (SARS-CoV-2) has resulted in unprecedented disruption to all businesses including the healthcare industry. Around the world, countries have declared states of emergency, causing an overnight transformation in the delivery of dental services. NHS England has issued advice to curtail non-essential elective surgeries in hospitals in order to consolidate the workforce and to limit the need for members of the public to leave their homes [2, 3].

Due to government social distancing policies and in some cases unavoidable self-isolation requirements, society is now reliant more than ever on digital platforms such as websites and social media, to access information about the COVID-19 virus and its effects on other aspects of life. Previous studies regarding orthodontic websites have demonstrated poor compliance with the ‘*Guidance on advertising’* document published by the General Dental Council (GDC) [4, 5]. This guidance advises that content is regularly updated and reviewed to ensure it is relevant and accurate [5]. The GDC has a clear stance on effective communication with patients. With a daily changing landscape filled with information that is almost outdated as soon as it is published, overarching principles laid out by the GDC remain absolute. The internet and social media provide an invaluable resource for practices to do this. Table 1 shows the applicable standards with regards to communication with patients during the ongoing pandemic. The GDC stipulate that patients must be provided with clear information about arrangements for emergency care (including the out of hours arrangements) and must be able to have details that allow contact by their preferred method [6, 7].

**Table 1:** GDC Standards relevant to communication with patients during the COVID-19 pandemic [6, 7].

GDC Standard	Accompanying Text
1.3.3	You must make sure that any advertising, promotional material or other information that you produce is accurate and not misleading, and complies with the GDC's guidance on ethical advertising.
2.3.9	You must provide patients with clear information about your arrangements for emergency care including the out of hours arrangements.
2.3.10	You should make sure patients have the details they need to allow them to contact you by their preferred method.
2.3.11	You should provide patients with clear information about any referral arrangements related to their treatment.

The nature of operative dentistry means that healthcare professionals and patients are highly susceptible to contracting COVID-19 [2]. As a result of government guidance, all routine dentistry in the United Kingdom (UK), including the practice of orthodontics has been ceased. Many practices remain open providing telephone triage for dental emergencies and outside of working hours patients are often directed to the NHS 111 service. Orthodontic emergencies commonly range from broken and trauma-inducing wires, through to debonded brackets and lost devices. These may result in pain, discomfort and be detrimental to the overall treatment plan. Many of these emergencies can be avoided and can also be partially managed at home with simple solutions.

At the time of writing, there has been very little guidance on the standard of information that should be distributed to patients in this turbulent time. The British Orthodontic Society (BOS) has provided regularly updated resources for practitioners to appropriately manage their services and effectively triage and advise patients on issues regarding orthodontic emergencies. Whether this information has been widely adopted is uncertain [8].

A questionnaire in the United States of America (USA) identified that 76% of orthodontists used social media for some of their practice marketing, and 89% of patients/parents used social media to gain information [9]. During this current climate, using these resources will be even more vital for keeping patients fully informed. Patients will be looking elsewhere for information and advice on the provision of future services and in emergency situations. Using websites and/or social media to relay this information is therefore a highly valuable strategy to ensure we are providing the highest duty of care. The use of alternative methods during this period will be necessary and is likely to require the use of teledentistry. This involves the delivery of dental care at a distance, using information and communication technologies.

Therefore, the aim of this paper is to evaluate the accessibility and quality of information on orthodontic emergencies provided by primary care specialist orthodontic practices in London during the COVID-19 pandemic. In what follows, we discuss ways in which websites, social media and other digital tools can be used as effective responses to COVID-19. By doing this, it is hoped practitioners will be able to learn from the best providers about how to remotely communicate with patients effectively, such that as many patients as possible can benefit.

## 2 Aims and objectives

To evaluate the information provided by primary care specialist orthodontic practices in London during the COVID-19 pandemic. This will be measured against defined criteria on websites and Facebook pages. In addition, any novel approaches to the delivery of information will be recorded and discussed.

## 3 Methodology

### 3.1 Standards

A service evaluation was conducted using the GDC *Standards* and GDC *Guidance on advertising* in combination with the BOS *Advice for Orthodontic Practices in Primary Care Settings* webpage [6, 7, 12]. Orthodontic practices were evaluated on whether their practice websites and Facebook pages cover the following points:

• Does the practice website include the practice name, address, contact number and email address?
• Has a COVID-19 update been provided?
• Has the practice provided contact information for emergencies (phone or email)?
• Are there contact details for out-of-hours emergencies?
• Does the practice provide any advice for managing emergencies at home?
• Is there any information regarding the cancellation of appointments or provision of future appointments?
• Does the practice reinforce the importance of, or provide, oral hygiene information?
• Does the practice state that it provides virtual appointments?
• Are there any novel approaches using information technology in the delivery of advice to patients?

### 3.2 Sample and data source

All providers of dental care in England are required by law, since 1^st^ April 2011, to be registered with the Care Quality Commission (CQC) and demonstrate that they continue to meet essential standards [10]. The CQC publish a publicly available, weekly register, of all premises providing dental care across England. The CQC website was accessed on 20th April 2020 to develop a list of practices (https://www.cqc.org.uk/what-we-do/services-we-regulate/find-dentist). Development of the practice list involved including only the results categorised as providing dental care located in the London region. Practices were subsequently truncated to those including the root term ‘ortho∗‘ within the practice name. Eighty-eight results were identified. Practices were excluded if no further practice details could be identified from the CQC list. Of the remaining 83 practices, the corresponding websites and Facebook pages were identified, Table 2. On reviewing these sources, they were then analysed and evaluated using the set criteria, Tables 3, 5, and 6.

**Table 2:** Practices included for service evaluation.

Total practices included	83
Website identified	78
Facebook identified	62
Practices without website or Facebook	4

## 4 Results

All data collected on the adherence to the GDC guidance were compiled, Table 3. A total of 83 practice were included in this study. There were 18 practices (23.1%) that did not comply with the GDC *Guidance on ethical advertising* document. This was wholly a result of 18 practices not providing the practice email address, which would be expected from most businesses and is a necessary requirement from the GDC.

**Table 3:** Website compliance with GDC *Guidance on advertising*.

Of the websites identified, do they display the following compulsory information?	Yes (N)	%
Practice name	78	100
Practice address	78	100
Practice phone	78	100
Practice email	60	76.9
Compliance with GDC guidance	60	76.9

The standards, based on GDC guidance and BOS advice, were used to scrutinize all practices for the remainder of the service evaluation. Seventeen practices (20.5%) that did not provide any update on either their website or Facebook page in relation to the COVID-19 pandemic; nearly three quarters (74.7%) provided a response by at least one of the platforms and over a quarter of practices (25.7%) provided information on both platforms (Table 4).

**Table 4:** Provision of updated information in response to the COVID-19 pandemic.

Of the practices identified, do they provide any updated information in response to the COVID-19 pandemic?	Yes (N)	%
No response given	17	20.5
Response given via Facebook or website	62	74.7
Response given via both Facebook and website	21	25.7

**Figure 1:**
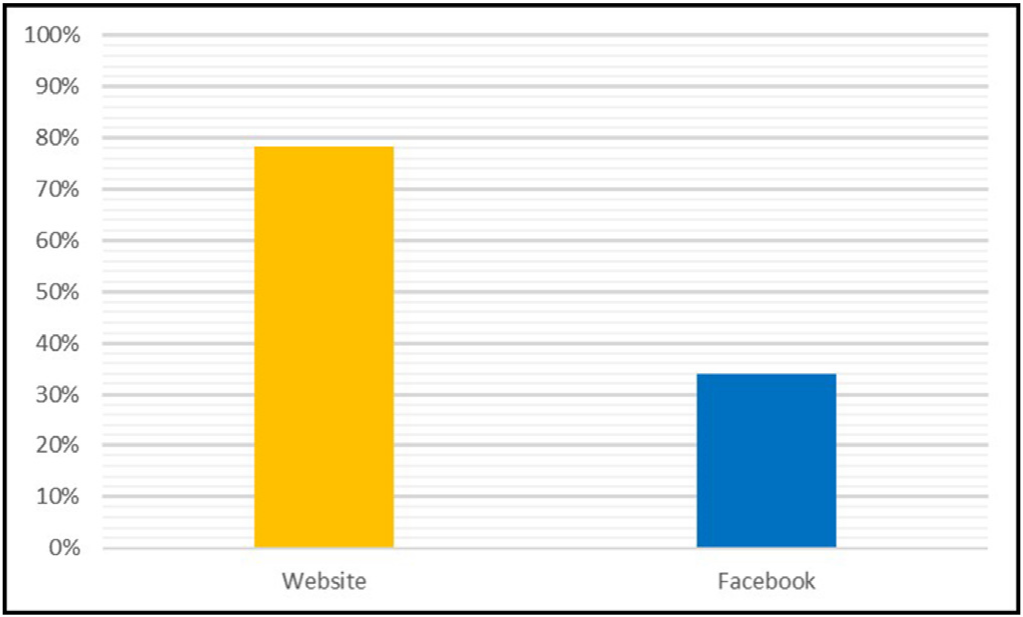
Comparison of orthodontic practices in London region providing an COVID-19 update.

It was more common for orthodontic practice websites to provide an update in response to COVID-19 in comparison to Facebook pages (Figure 1). Of the 78 websites, 78.2% provided an update in response to COVID-19, compared with 33.9% of the 62 Facebook pages.

**Figure 2:**
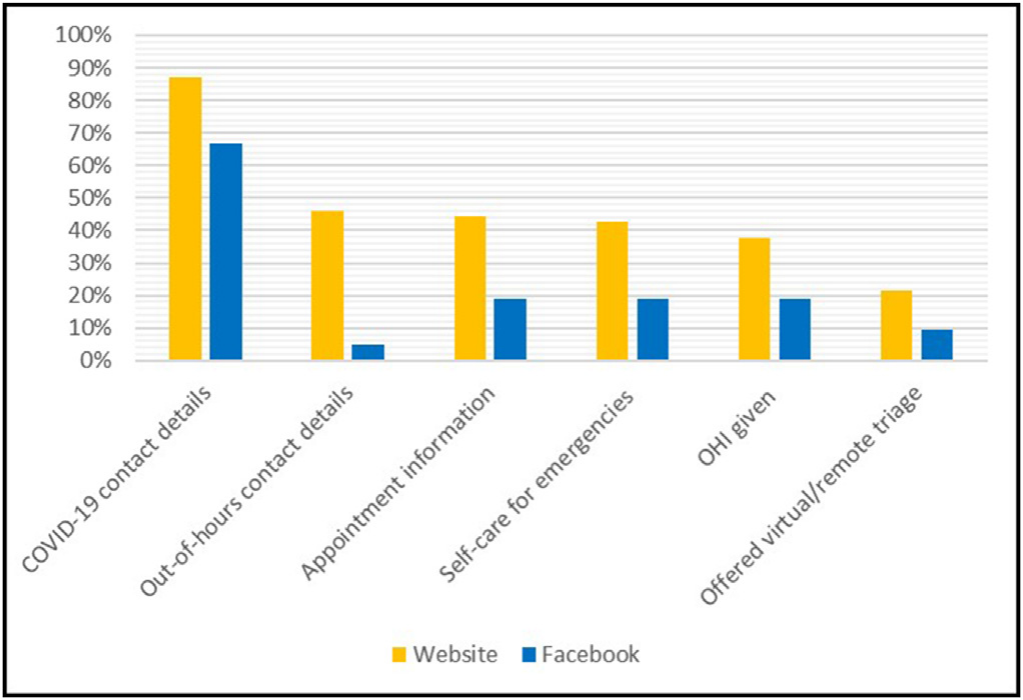
Comparison of orthodontic practices in the London region in their level of content provided in a COVID-19 update.

Of the websites providing updates, 86.9% contained relevant contact details for patients during the COVID-19 pandemic. However, only 46.0% provided details for patients outside of normal hours. A similar amount provided information about the process of rescheduling existing appointments and future appointments and information regarding management of orthodontic emergencies at home; 44.3% and 42.6% respectively. Fewer (37.8%) provided advice or reinforced good oral hygiene habits (Tables 5 and 6) (Figure 2).

**Table 5:** Content provided by practice website pages in COVID-19 updates.

Of the websites identified providing a response to COVID-19, do they display the following information?	Yes (N)	%
Provided contact information for emergencies during COVID-19 pandemic	53	86.9
Provided contact details for out-of-hours emergencies	28	45.9
Information provided on the cancellation of appointments or provision of future appointments	27	44.2
Provided any advice for managing emergencies at home	26	42.6
Provide any oral hygiene information	23	37.7
Offering virtual/remote appointments	13	21.3

**Table 6:** Content provided by practice Facebook pages in COVID-19 updates.

Of the Facebook pages identified providing a response to COVID-19, do they display the following information?	Yes (N)	%
Provided contact information for emergencies during COVID-19 pandemic	14	66.7
Provided contact details for out-of-hours emergencies	1	4.8
Information provided on the cancellation of appointments or provision of future appointments	4	19.0
Provided any advice for managing emergencies at home	4	19.0
Provide any oral hygiene information	4	19.0
Offering virtual/remote appointments	2	9.5

The subsequent analysis of the orthodontic practice Facebook pages generated a lower volume of relevant information in response to the COVID-19 pandemic. Of the 21 Facebook pages identified that contained COVID-19 updates, 66.7% included contact details for patients, however, only 4.8% provided contact details for patients outside of normal hours. Of the Facebook pages, 19% provided information about the process of rescheduling existing appointments and future appointments, management of orthodontic emergencies and provided oral hygiene information (Table 5).

Interestingly, 13 practices (15.6%) offered services to provide virtual emergency appointments. Of these; two provided this on both their website and social media page. The BOS provide information on how to successfully implement this. When considering other novel strategies beyond the search criteria, it was exciting to see that seven practices were also offering video consults for new patients. Two practices had also produced their own videos in order to enable patients to manage emergencies from home.

## 5 Discussion

COVID-19 presents what is arguably going to be the greatest test to medicine and dentistry of our generation. In the modern era, websites and social media provide a useful means to aid dissemination of crucial information to new patients, existing patients as well as allowing for providers to advertise their services and carry out direct marketing [11].

All routine care including orthodontics saw an immediate decline and eventual cessation following guidance from England's Chief Dental Office (CDO), issued on 25^th^ March 2020, that all non-urgent care should be stopped [12]. The COVID-19 pandemic presents an unprecedent period in the history of healthcare in the United Kingdom.

With the number of adults seeking orthodontic treatment in the UK continuing to rise and in excess of 200,000 children and teenagers in England and Wales undergoing treatment within the NHS every year, the need for effective communication of orthodontic advice is more critical than ever [13].

**Figure 3:**
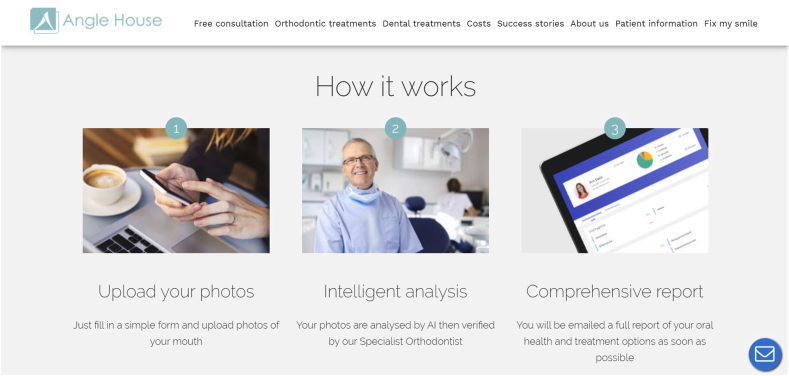
An example of how practices are offering novel ways to offer new patient consultations. Available at https://www.anglehouseorthodontics.co.uk/smile-mate/. Permission obtained from Angle House Orthodontics.

It is understandable therefore that many practices were neither well equipped nor prepared for what ensued in the days following the CDO's guidance. The safety and suitability of remote consulting – i.e. teledentistry has been an area for which dental defence societies have been inundated with queries in the last few weeks [14]. With such uncertainty regarding teledentistry and without standardised guidance, it can be no surprise that there is considerable variation in the quality of its delivery (Figure 3). The main facet here, is that the overarching GDC Standards should still apply.

**Figure 4:**
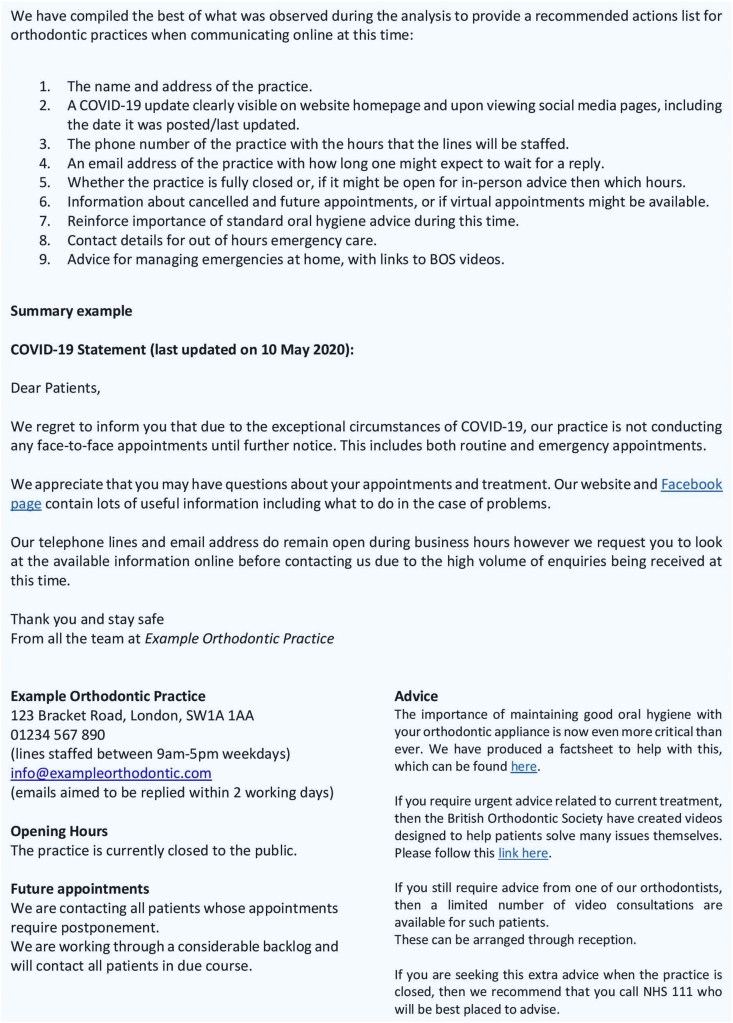
Recommendations compiled from observed best practice, with a summary example.

Whilst our study results do indeed show huge variation in the delivery of remote information, the best-practice discovered by this study and highlighted in our recommendations in Figure 4, will be working together towards better care for patients and practice alike. By reducing time waste for patients not having to hunt for reliable information, as well as the ability to have this information without a time restraint, effectively reduces the amount of time spent by practice staff and clinicians during working hours and can better prioritise true orthodontic emergencies rather than repeat the same information over the phone.

Initial dental planning conversations bubbled around personal protective equipment (PPE), volunteering and redeployment. The New York Times reminded the world that dentistry had the highest risk of COVID-19 of any profession across the planet [15]. As of yet, no published exit-strategy for COVID-19 exists for the UK. We are seeing the government gradually relax some restrictions but remain unaware of how long the pandemic will continue to halt routine dentistry and orthodontics. What we do know is that considerable disruption is expected to continue across all non-essential medical services for months to come. It should therefore be incumbent upon practices providing specialist orthodontic services, to improve their online information for the huge number of patients currently under their care.

COVID-19 provides opportunities for practices to reflect on the information they already provide to their patients online and how this then might be improved upon. Recent research into the design and content of orthodontic practice websites in the UK revealed wide variation amongst websites and suboptimal performance against validated measures of accessibility, usability and reliability [11]. This echoes results of a recent Dutch study which found that the informative value and design of orthodontic practice websites in The Netherlands was low and information was set above the average reading age of the population [16]. As we move forward, even as we possibly exit the disruption of COVID-19, It would be encouraging to see patients able to access relevant and reliable information from the website or social media page(s) of the practice they attend.

**Figure 5:**
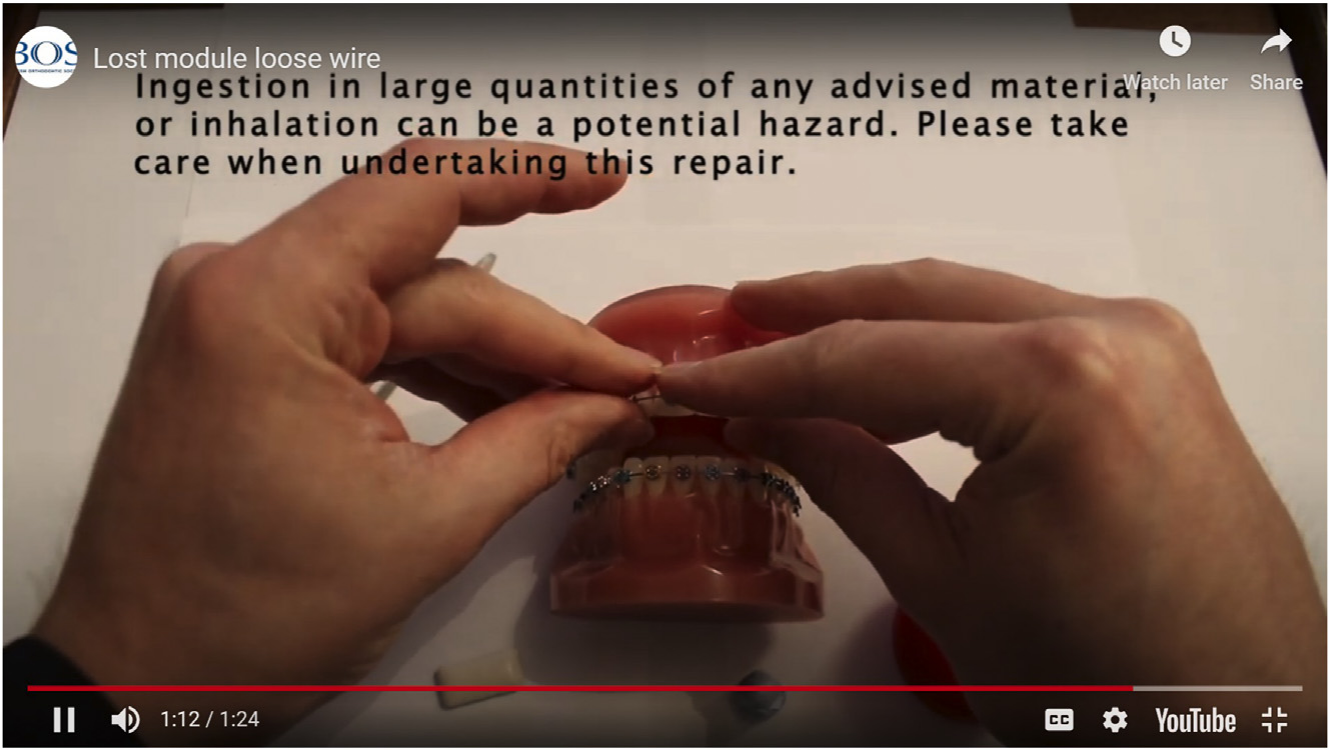
A number of videos have been produced by the BOS demonstrating how patients can access advice to manage problems at home. Available at https://www.youtube.com/watch?v=VG3t6HfTOZo. Permission obtained from the British Orthodontic Society.

In Figure 4 we have complied the areas of best practice observed during this research. The authors contend that if primary care specialist orthodontic practices were to follow these recommendations, they would be doing their patients a huge service by providing online, relevant and reliable information effectively, during these uncertain times. This would provide a blanket format of response for delivery information across websites and social media. Practices could use easy-to-follow videos like that shown in Figure 5; one of a number of videos demonstrating how patients can access advice to manage problems at home.

Following traditional face-to-face consultations, the internet is the second most popular source of healthcare information for patients [4, 17]. With 95.2% of primary care specialist orthodontic practices in this study possessing a website and/or a social media account, this reinforces the need for alternative methods of effective online communication when conventional face-to-face options are not available. Emergencies are unpredictable, and when they happen outside normal working hours, fully accessible and relevant information will benefit all patients. Whilst not the remit of this study to investigate non-digital means of practice communication, it is true that letters and telephone calls are indeed longstanding, effective communication tools. It could well be that these practices posted information sheets to patients, however sending out mailshots means that not all patients can be contacted at the same time. This has the potential of some patients receiving updates long before their fellow patients. This could cause unnecessary concern from those yet to be contacted. Given the rapidly changing nature of public guidance regarding the pandemic, however, there a number of perceived issues which mean online communication tools are crucial in situations such as this. Whilst 93% of households have internet access [18], when considering the younger patient demographic of adult orthodontics [19], it might be more appropriate to look at those aged 16–44 years for which daily internet access stands at 99%. This demonstrates the near universality of internet access in the patient and parent population [18]. Finally, where a process involves extensive use of contacting individual patients by e.g. telephone, this will take considerable labour from practice staff and is likely to mean busy phone lines hindering those with urgent need of advice from being able to access that.

London is home to one of the most ethnically diverse populations in the world [20], and as such the authors feel the results of this study are applicable and generalisable to all primary care specialist orthodontic practices in the United Kingdom. Novel approaches such as teledentistry may change the way we work not only now but, in the months and years which follow [21]. Whether in medicine, dentistry or other healthcare professions, with smartphone use now globally ubiquitous, all patients need is an internet connection and a camera phone to allow them to talk to a clinician [22]. As a second wave of COVID-19 is plausible, it seems prudent for dental organisations to embrace models of mobile and remote dentistry. Engaging digital means of communication alongside the non-digital, will enable providers to adapt to the rapidly, ever-changing needs of society.

## 6 Limitations

- The sampling method used did not account for practices that did not include ‘ortho∗’ within the practice name. Hence there may be other practices that are providing services not included within our sample.
- It may be that practices simply didn't mention on their websites that they have remote/virtual triaging available online. However, with nearly three-quarters of the practices in the study providing updated COVID-19 information, this would not be seen as a major limitation to the study. The authors contend the presence/absence of virtual appointments should be mandatory information provided by a practice who cannot offer face-to-face appointments during the current pandemic
- As previously alluded to, practices may be contacting patients by other means e.g. information by post. Practices may also have done a certain amount of information sharing when it was required to cancel/rearrange upcoming appointments following the CDO's guidance. The GDC do, however, state that accessing information should be about patients being able to do it in the form they desire, and it would be natural to assume with social distancing guidelines in operation, the first place many people would look would be a practice website or social media page
- The COVID-19 pandemic is fast-paced and ever changing. More practices may now be advertising virtual consultations or other online information as a result. The authors are of the opinion, however, that the information contained within this research has strong merit even against this limitation
- Unfortunately, our data collection did not collect data about whether they were NHS or private providers and hence we were unable to analyse if one category of provider was able to communicate more effectively online than the other.

## 7 Conclusions

In summary, the majority of orthodontic practices were providing updated information in response to the COVID-19 pandemic online. Unfortunately, not all complied with the GDC advertising criteria. It is important for websites to include accurate and contemporaneous information. Websites and social media are two of a larger number of ways that can be used to effectively communicate with patients, each with their own merits. We have outlined what we believe to be best practice for ensuring patients are provided with accurate and clear information. It is hoped that this paper will bring attention to, and raise awareness of, digital means to help to improve the quality of information made available to the public.

## Declarations

### Author contribution statement

J. Woolley, C. Donnell, S. Worthington: Conceived and designed the experiments; Performed the experiments; Analyzed and interpreted the data; Contributed reagents, materials, analysis tools or data; Wrote the paper.

### Funding statement

This research did not receive any specific grant from funding agencies in the public, commercial, or not-for-profit sectors.

### Declaration of interests statement

The authors declare no conflict of interest.

### Additional information

No additional information is available for this paper.
